# The Italian lichens dataset from the TSB herbarium (University of Trieste)

**DOI:** 10.3897/BDJ.11.e96466

**Published:** 2023-02-27

**Authors:** Matteo Conti, Pier Luigi Nimis, Mauro Tretiach, Lucia Muggia, Andrea Moro, Stefano Martellos

**Affiliations:** 1 Dept. Of Life Sciences, University of Trieste, Trieste, Italy Dept. Of Life Sciences, University of Trieste Trieste Italy

**Keywords:** collection, diversity, georeference, occurrence, specimens

## Abstract

**Background:**

The "Herbarium Universitatis Tergestinae" (TSB), with a total of ca. 50,000 specimens, includes the largest modern collection of lichens in Italy, with 25,796 samples collected from all over the country since 1984, representing 74% of all taxa known to occur in Italy. Almost all specimens have been georeferenced “a posteriori”. The dataset is available through GBIF, as well as in ITALIC, the Information System of Italian Lichens.

**New information:**

The TSB Herbarium hosts the largest modern lichen collection in Italy, with a total of ca. 50,000 specimens. This dataset contains all of the 25,796 specimens collected within the administrative borders of Italy. Amongst them, 98% are georeferenced and 87% have the date of collection. The dataset includes several type specimens (isotypes and holotypes) and exsiccata.

## Introduction

Herbaria are an important source of falsifiable biodiversity data; stored specimens can be used to validate observations (Willis et al. 2017), to provide data for the assessment of Red Lists (Callmander et al. 2005, Nascimbene et al. 2012), to obtain DNA for answering questions of evolution, genetic diversity etc. (Taylor and Swann 1994) and to depict the presence of a taxon in a specific space and time. Geo-referenced data obtained from specimens can be used in distribution modelling and biogeographic studies to assess the past extent of a taxon (Marsico et al. 2020, Albani Rocchetti et al. 2021), to depict its current distribution and to predict potential range shifts in a global changes scenario (Loiselle et al. 2008, Attorre et al. 2018, Meineke et al. 2018, Lang et al. 2019).

The TSB Herbarium hosts the largest modern lichen collection in Italy, with a total of ca. 50,000 specimens. It was the first lichen collection in Italy digitised in a database (Nimis 1990) using the software described by Lagonegro et al. (1982). The Italian collection, started in 1984, contains 25,796 specimens from all parts of the country, mainly resulting from large field surveys, such as those in Sardinia (Nimis and Poelt 1987), eastern Peninsular Italy (Nimis and Tretiach 1999), western Peninsular Italy (Nimis and Tretiach 2004) and in several small islands and protected areas (e.g. Nimis (1985), Nimis et al. (1990), Nimis et al. (1994), Nimis et al. (1996)). The Italian collection hosts also several exsiccata: "Erbario Crittogamico Italiano" (Società Crittogamologica Italiana) (415 specimens), A. Vĕzda "Lichenes Selecti Exsiccati" (75), A. Vĕzda "Lichenes Rariores Exsiccati" (54), "Lichenes Italici Exsiccati" (Società Lichenologica Italiana) (35) "Plantae Graecenses" (Karl-Franzens-Universität Graz) (8) etc. and duplicates from other herbaria: Herbarium CLU (427), Herbarium PA (29), Herbarium Museum Caffi (25), Herbarium MOD (15), Herbarium Zirnich (8) etc.

Before the publication of the TSB Herbarium dataset, querying the GBIF (2022) for lichen occurrences in Italy returned about 12,000 records, none of which came from an Italian herbarium, a number which is quite small if compared to those of several others European countries, for example, up to 2 million records for the UK.

In the framework of project "Dryades" (Nimis et al. 2003), an effort to aggregate data from Italian lichen collections is being carried out, aiming at making data available online on ITALIC, the information system on Italian lichens (Nimis 2022). At the same time, records will be encoded in the Darwin Core standard (Wieczorek et al. 2012) and will be shared in the GBIF. In Italy, there are several important historical collections, mostly dating back to the “Golden Period” of Italian Lichenology in the second half of the 19^th^ century (Nimis 2018), such as the herbaria of A.B. Massalongo (VER), F. Baglietto (MOD), M. Anzi (TO) and A. Jatta (NAP) (Tretiach and Valcuvia Passadore 1990). While efforts for their digitisation are foreseen, they will provide serious challenges, both as far as nomenclature and the georeferencing of localities are concerned. The latter is an especially challenging task, since localities are reported with obsolete toponyms or not reported at all. Thus, we prioritised 13 modern herbaria (with specimens collected after 1950): CLU, FI, GDOR, GE, HLUC, ORO, SI, TO, TSB and the private herbaria of G. Gheza, J. Nascimbene, S. Ravera and W. von Brackel. The digitisation and publication of the TSB lichen collection is, thus, the first step towards making all the data from Italian lichen collections publicly available.

## Sampling methods

### Study extent

The Italian collection of the TSB lichen herbarium hosts specimens collected from all the 20 administrative regions of Italy.

### Sampling description

Specimens were mostly gathered in the course of field surveys devoted to the exploration of different areas of the country, where both common and rare species were collected. All specimens are stored in 15 cm x 10 cm paper envelopes. Label data were digitalised and stored in a MySQL database, which has been made publicly available on ITALIC, the information system on Italian lichens (Nimis 2022) and on GBIF (Martellos et al. 2022).

### Quality control

Specimens were collected and identified by experienced lichenologists (mostly by Nimis PL, Tretiach M and Muggia L), and sometimes revised by foreign specialists. Scientific names have been automatically aligned to the latest checklist of Italian lichens (Nimis 2016) by means of a customised version of the FlorItaly name matching tool (Conti et al. 2021). The verbatim scientific name, i.e. the name originally written on the label, has been retained together with the currently accepted name. Since for almost all specimens geographical coordinates of the collection locality were missing, all specimens were georeferenced a posteriori using Google Maps, Google Earth and regional GIS maps. The georeferencing process followed the best practices by Chapman and Wieczorek (2020).

## Geographic coverage

### Description

**Figure 1.**
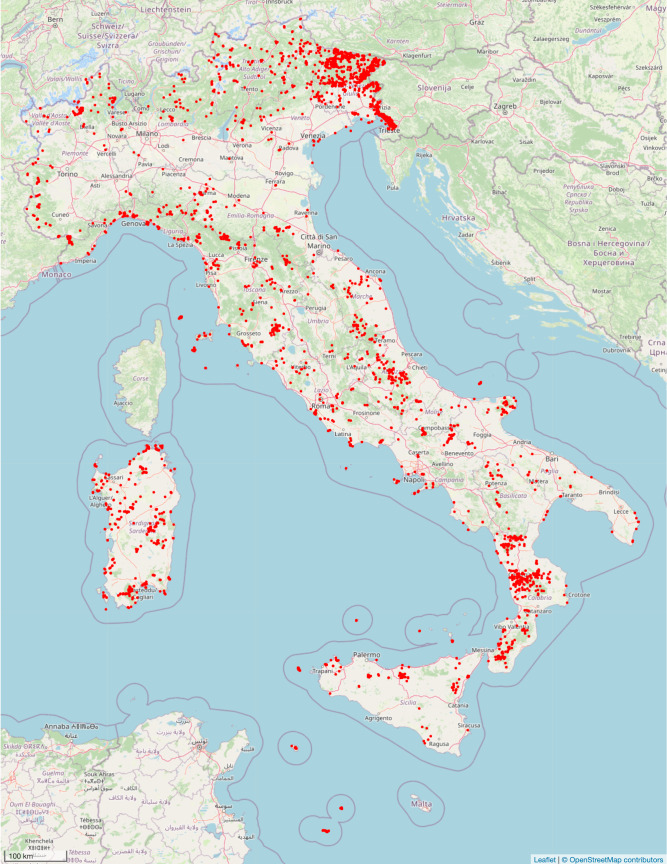
Distribution map of TSB herbarium specimens in Italy; created with Leaflet (Agafonkin 2022).

 The dataset contains specimens collected in all the 20 administrative regions of Italy: Abruzzo (1451), Basilicata (830), Calabria (1503), Campania (888), Emilia Romagna (842), Friuli Venezia Giulia (6235), Lazio (998), Liguria (567), Lombardia (193), Marche (1098), Molise (598), Piemonte (1904), Puglia (1415), Sardegna (2631), Sicilia (1595), Toscana (1930), Trentino Alto Adige (314), Umbria (97), Valle d’Aosta (148), andVeneto (532). Only for 27 specimens the locality of collection was not reported in the database. The distribution of specimens in the Italian territory is shown in Fig. 1.

### Coordinates

35.317 and 49.668 Latitude; 6.284 and 18.809 Longitude.

## Taxonomic coverage

### Description

The specimens included in the dataset, according to the GBIF Taxonomic Backbone, belong to 44 orders, 118 families and 459 genera.

The following families are represented: Abrothallaceae, Acarosporaceae, Adelococcaceae, Aphanopsidaceae, Arctomiaceae, Arthoniaceae, Arthopyreniaceae, Arthrorhaphidaceae, Baeomycetaceae, Biatorellaceae, Bionectriaceae, Caliciaceae, Candelariaceae, Cantharellaceae, Carbonicolaceae, Catillariaceae, Chrysotrichaceae, Cladoniaceae, Coccocarpiaceae, Coenogoniaceae, Collemataceae, Coniocybaceae, Cystocoleaceae, Dacampiaceae, Dactylosporaceae, Dermateaceae, Fuscideaceae, Gloeoheppiaceae, Gomphillaceae, Graphidaceae, Gyalectaceae, Haematommataceae, Helocarpaceae, Herpotrichiellaceae, Hygrophoraceae, Hymeneliaceae, Hysteriaceae, Icmadophilaceae, Koerberiaceae, Lecanographaceae, Lecanoraceae, Lecideaceae, Leprocaulaceae, Leptosilliaceae, Lichenoconiaceae, Lichenotheliaceae, Lichinaceae, Lichinodiaceae, Lobariaceae, Lopadiaceae, Massalongiaceae, Megasporaceae, Melaspileaceae, Microcaliciaceae, Monoblastiaceae, Mycocaliciaceae, Mycoporaceae, Mycosphaerellaceae, Mytilinidiaceae, Naetrocymbaceae, Nectriaceae, Nephromataceae, Niessliaceae, Nitschkiaceae, Ochrolechiaceae, Opegraphaceae, Ophioparmaceae, Pannariaceae, Parmeliaceae, Patellariaceae, Peltigeraceae, Peltulaceae, Pertusariaceae, Phaeococcomycetaceae, Phlyctidaceae, Physciaceae, Pilocarpaceae, Placynthiaceae, Pleomassariaceae, Pleosporaceae, Polycoccaceae, Porinaceae, Porpidiaceae, Protothelenellaceae, Psilolechiaceae, Psoraceae, Pycnoraceae, Pyrenidiaceae, Pyrenulaceae, Ramalinaceae, Ramboldiaceae, Rhizocarpaceae, Roccellaceae, Roccellographaceae, Sagiolechiaceae, Sarrameanaceae, Schaereriaceae, Scoliciosporaceae, Sphaerophoraceae, Sphinctrinaceae, Sporastatiaceae, Stereocaulaceae, Stictidaceae, Strangosporaceae, Strigulaceae, Teloschistaceae, Tephromelataceae, Teratosphaeriaceae, Thelenellaceae, Thelocarpaceae, Trapeliaceae, Trypetheliaceae, Tympanidaceae, Umbilicariaceae, Vahliellaceae, Verrucariaceae, Xanthopyreniaceae and Xylographaceae.

**Figure 2.**
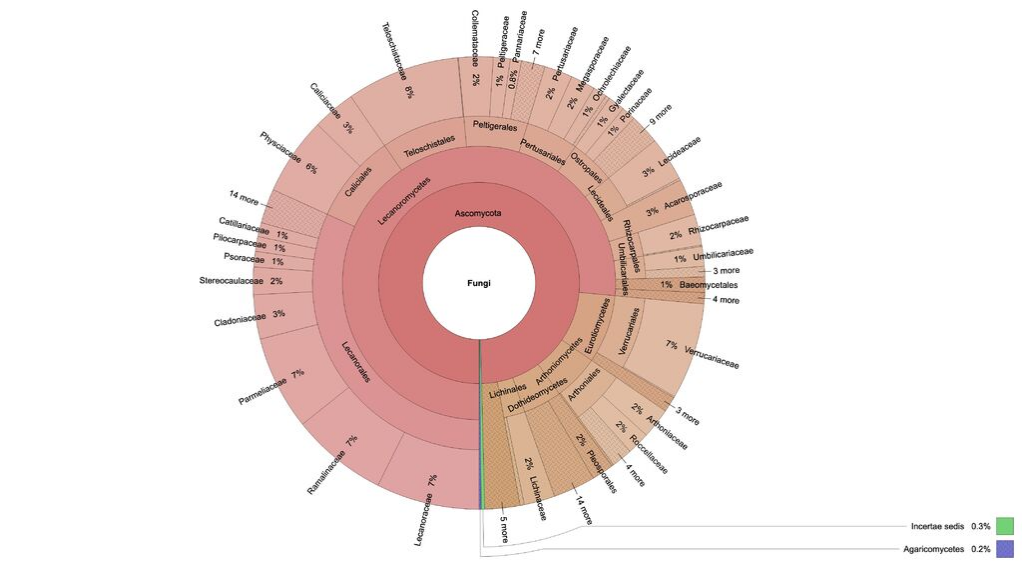
Taxa distribution between classes, orders and families, created using krona graph tool (Ondov et al. 2011).

Taxa and specimens numbers for each kingdom, phylum, class, order, family and genus are available in a spreadsheet (Suppl. material 1) and can be graphically visualised as a krona graph (Fig. 2; the interactive file is provided in Suppl. material 2).

## Temporal coverage

### Notes

**Figure 3.**
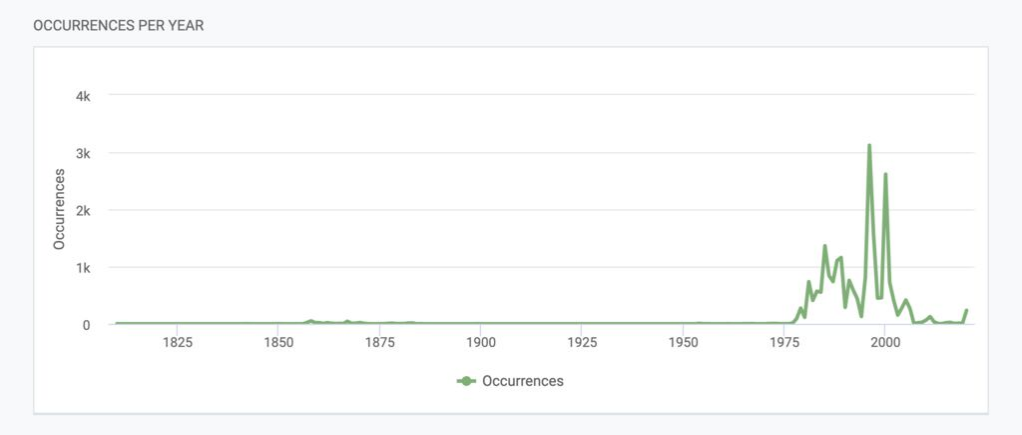
Lichens occurrences per year.

Specimens have been collected and recorded from 1810 to 2021. Occurrences per year are shown in Fig. 3. All specimens dated before 1984, the year in which the TSB collection was started, come from exsiccata collections or from exchanges with other Herbaria. The highest number of accessions was between 1987 and 2010, corresponding to the peak of lichenological exploration of Italy by researchers of the University of Trieste.

## Usage licence

### Usage licence

Creative Commons Public Domain Waiver (CC-Zero)

### IP rights notes

This work is licensed under a Creative Commons Attribution (CC-BY) 4.0 License.

## Data resources

### Data package title

TSB Lichen Herbarium

### Resource link

https://www.gbif.org/dataset/859c6946-f762-11e1-a439-00145eb45e9a

### Alternative identifiers

https://doi.org/10.15468/dl.t3kjr9

### Number of data sets

1

### Data set 1.

#### Data set name

TSB Lichen Herbarium

#### Data format

Darwin Core

#### Download URL

https://www.gbif.org/occurrence/download?dataset_key=859c6946-f762-11e1-a439-00145eb45e9a

#### Description

This is the largest modern lichen collection of speciemens collected within the administrative borders of Italy. It was started in 1984 and, to date, it includes ca. 26,000 samples, collected mainly by P.L. Nimis, M. Tretiach and L. Muggia (Martellos et al. 2022).

**Data set 1.** 

Column label	Column description
occurrenceID	An identifier for the Occurrence.
institutionID	An identifier for the institution having custody of the object.
institutionCode	The acronym in use by the institution having custody of the object (TSB for all specimens).
basisOfRecord	The specific nature of the data record (PreservedSpecimen for all specimens).
catalogNumber	An identifier for the record within the dataset or collection.
recordedBy	A list of names of people, groups or organisations responsible for recording the original Occurrence.
occurrenceRemarks	Comments or notes about the Occurrence.
eventDate	The date-time or interval during which an Event occurred.
year	The four-digit year in which the Event occurred, according to the Common Era Calendar.
continent	The name of the continent in which the Location occurs (Europe for all specimens).
country	The name of the country or major administrative unit in which the Location occurs (Italy for all specimens).
countryCode	The standard code for the country in which the Location occurs (IT for all specimens).
stateProvince	The name of the next smaller administrative region than country (state, province, canton, department, region etc.) in which the Location occurs.
locality	Description of the place were the specimen was taken.
minimumElevationInMeters	The lower limit of the range of elevation in metres.
maximumElevationInMeters	The upper limit of the range of elevation in metres.
decimalLatitude	The latitude in decimal degrees. Locations were georeferenced a posteriori according on the information written on the label.
decimalLongitude	The longitude in decimal degrees. Locations were georeferenced a posteriori according on the information written on the label.
geodeticDatum	The ellipsoid, geodetic datum or spatial reference system (SRS) upon which the geographic coordinates given in decimalLatitude and decimalLongitude are based (WGS84 for all specimens).
coordinateUncertaintyInMetres	The horizontal distance from the given decimalLatitude and decimalLongitude describing the smallest circle containing the whole of the Location.
scientificName	The full scientific name, with authorship. Assigned according to the Italian checklist of lichens.
verbatimIdentification	A string representing the taxonomic identification as it appeared in the original record.
typeStatus	The nomenclatural type applied to the subject.
kingdom	The full scientific name of the kingdom in which the taxon is classified.
phylum	The full scientific name of the phylum or division in which the taxon is classified.
class	The full scientific name of the class in which the taxon is classified.
order	The full scientific name of the order in which the taxon is classified.
family	The full scientific name of the family in which the taxon is classified.
taxonRank	The taxonomic rank of the most specific name in the scientificName.
licence	A legal document giving official permission to do something with the resource.
type	The nature or genre of the resource (PhysicalObject for all specimens).
language	Thelanguage of the resource.

## Supplementary Material

6399D856-8454-5D27-8032-14642F91CAA4 10.3897/BDJ.11.e96466.suppl1 Supplementary material 1 Taxa and specimens table Data type table Brief description A table showing the total number of taxa and specimens in the dataset. File: oo_757350.txt https://binary.pensoft.net/file/757350 Matteo Conti

CB2E719A-EF05-5919-B3BF-DC3596FA8079 10.3897/BDJ.11.e96466.suppl2 Supplementary material 2 Krona graph taxa and specimens Data type html file Brief description A Krona graph showing taxa and specimens in the herbarium. File: oo_757351.html https://binary.pensoft.net/file/757351 Matteo Conti
