# Supplementary material for: The Italian lichens dataset from the TSB herbarium (University of Trieste)
Source: Biodivers Data J. 2023 Feb 27;11:e96466. doi: 10.3897/BDJ.11.e96466 (PMC10848505; doi:10.3897/BDJ.11.e96466)
Supplement: Supplementary material 2 — Krona graph taxa and specimens [file bdj-11-e96466-s002.html]

Javascript must be enabled to view this page.

magnitude

TSB taxa
TSB specimens

 1990
 25796

 1990
 25796

 1982
 25741

 108
 1048

 105
 1038

 43
 362

 35
 324

 1
 3

 2
 15

 2
 6

 1
 2

 1
 11

 1
 1

 3
 41

 3
 41

 11
 109

 7
 93

 2
 9

 1
 1

 1
 6

 15
 106

 1
 1

 13
 104

 1
 1

 32
 417

 3
 24

 2
 35

 6
 88

 1
 9

 4
 22

 2
 12

 1
 6

 1
 8

 1
 55

 2
 3

 1
 34

 1
 1

 4
 99

 2
 16

 1
 5

 1
 3

 1
 3

 3
 10

 3
 10

 2
 9

 1
 1

 18
 477

 18
 477

 16
 475

 1
 88

 15
 387

 2
 2

 2
 2

 14
 93

 14
 93

 14
 93

 12
 84

 2
 9

 91
 456

 1
 2

 1
 2

 1
 2

 12
 22

 1
 4

 1
 4

 10
 15

 1
 1

 2
 4

 1
 1

 6
 9

 1
 3

 1
 3

 3
 6

 3
 6

 1
 4

 2
 2

 6
 8

 6
 8

 3
 3

 3
 5

 8
 35

 8
 35

 1
 6

 6
 25

 1
 4

 1
 36

 1
 36

 1
 36

 1
 1

 1
 1

 1
 1

 3
 7

 3
 7

 3
 7

 1
 3

 1
 3

 1
 3

 8
 123

 8
 123

 6
 116

 2
 7

 1
 1

 1
 1

 1
 1

 1
 12

 1
 12

 1
 12

 31
 160

 5
 32

 5
 32

 7
 27

 2
 21

 1
 4

 4
 2

 2
 4

 1
 3

 1
 1

 1
 3

 1
 3

 12
 90

 5
 5

 6
 59

 1
 26

 1
 1

 1
 1

 2
 2

 2
 2

 1
 1

 1
 1

 8
 31

 8
 31

 1
 5

 2
 3

 5
 23

 6
 9

 5
 8

 1
 3

 4
 5

 1
 1

 1
 1

 161
 1223

 2
 3

 1
 1

 1
 1

 1
 2

 1
 2

 12
 41

 9
 27

 5
 10

 1
 3

 2
 11

 1
 3

 3
 14

 3
 14

 8
 42

 8
 42

 1
 3

 1
 8

 6
 31

 139
 1137

 1
 1

 1
 1

 1
 3

 1
 3

 137
 1133

 4
 44

 8
 152

 10
 68

 1
 1

 7
 94

 6
 27

 4
 54

 2
 2

 1
 1

 1
 1

 5
 34

 2
 125

 3
 25

 2
 4

 1
 29

 2
 24

 13
 48

 2
 3

 1
 1

 10
 45

 17
 57

 30
 263

 3
 8

 2
 23

 5
 10

 1
 3

 1
 3

 1
 3

 4
 7

 4
 7

 1
 2

 3
 5

 1522
 22168

 52
 323

 52
 323

 30
 134

 2
 5

 1
 1

 2
 5

 2
 21

 4
 9

 10
 147

 1
 1

 1
 8

 1
 8

 1
 8

 22
 199

 4
 34

 2
 5

 2
 29

 14
 126

 3
 31

 1
 2

 5
 40

 5
 53

 4
 39

 1
 15

 1
 1

 2
 23

 172
 2610

 61
 579

 3
 10

 2
 55

 24
 150

 11
 73

 1
 4

 1
 19

 2
 87

 8
 166

 2
 2

 1
 2

 6
 11

 111
 2031

 4
 142

 1
 10

 2
 22

 1
 89

 1
 6

 2
 16

 12
 287

 2
 18

 20
 642

 1
 35

 12
 387

 50
 337

 2
 24

 1
 16

 13
 68

 13
 68

 2
 2

 7
 44

 3
 3

 1
 19

 1
 2

 1
 2

 1
 2

 632
 10265

 1
 2

 1
 2

 4
 12

 3
 5

 1
 7

 2
 2

 2
 2

 20
 351

 11
 218

 1
 19

 8
 114

 63
 1137

 62
 1120

 1
 17

 10
 22

 1
 2

 9
 20

 2
 30

 2
 30

 1
 2

 1
 2

 4
 10

 1
 3

 1
 5

 1
 1

 1
 1

 147
 2160

 1
 6

 5
 30

 1
 1

 6
 148

 72
 1035

 20
 491

 7
 34

 16
 175

 1
 1

 2
 33

 10
 150

 1
 7

 1
 23

 1
 8

 2
 13

 1
 5

 134
 3114

 2
 24

 1
 5

 1
 7

 1
 24

 5
 74

 7
 143

 3
 22

 1
 26

 1
 4

 1
 3

 4
 159

 2
 222

 1
 7

 1
 4

 6
 165

 4
 11

 1
 14

 1
 24

 1
 34

 2
 26

 6
 282

 5
 137

 2
 12

 2
 3

 3
 59

 6
 319

 4
 256

 1
 5

 2
 59

 9
 153

 1
 3

 1
 48

 1
 115

 7
 64

 2
 26

 2
 134

 6
 122

 1
 10

 13
 71

 1
 3

 2
 52

 11
 183

 21
 82

 3
 8

 3
 7

 1
 1

 11
 63

 3
 3

 2
 11

 2
 11

 22
 274

 2
 4

 1
 3

 10
 158

 2
 7

 6
 101

 1
 1

 137
 1840

 2
 4

 18
 197

 3
 3

 1
 4

 9
 33

 5
 117

 1
 10

 2
 6

 2
 7

 1
 3

 3
 11

 21
 212

 2
 13

 2
 7

 7
 217

 31
 701

 7
 138

 15
 99

 5
 58

 1
 2

 1
 2

 2
 3

 2
 3

 8
 82

 8
 82

 2
 12

 2
 12

 39
 508

 1
 1

 21
 280

 9
 188

 8
 39

 2
 4

 2
 4

 8
 605

 2
 23

 1
 1

 5
 581

 66
 636

 63
 624

 5
 20

 3
 39

 1
 2

 4
 117

 6
 47

 2
 12

 1
 1

 28
 191

 1
 25

 10
 111

 1
 47

 1
 12

 2
 4

 2
 4

 1
 8

 1
 8

 87
 915

 2
 50

 2
 50

 5
 8

 1
 2

 1
 2

 2
 3

 1
 1

 14
 242

 8
 155

 2
 57

 2
 2

 1
 9

 1
 19

 24
 174

 17
 145

 2
 21

 5
 8

 1
 2

 1
 2

 2
 112

 2
 112

 20
 153

 11
 35

 7
 107

 1
 7

 1
 4

 4
 11

 4
 11

 1
 8

 1
 8

 8
 115

 1
 1

 1
 15

 2
 55

 2
 25

 2
 19

 6
 40

 1
 3

 1
 4

 4
 33

 123
 1932

 1
 2

 1
 2

 49
 864

 1
 13

 1
 16

 6
 165

 7
 151

 5
 184

 9
 78

 1
 6

 2
 8

 17
 243

 2
 12

 1
 9

 1
 3

 10
 161

 2
 79

 1
 19

 3
 53

 4
 10

 3
 17

 1
 13

 1
 2

 1
 2

 6
 107

 6
 107

 16
 211

 6
 79

 4
 22

 2
 28

 1
 42

 1
 20

 1
 11

 1
 9

 24
 426

 19
 346

 5
 80

 10
 121

 10
 121

 2
 11

 2
 11

 101
 1574

 4
 75

 1
 15

 1
 22

 2
 38

 35
 517

 12
 114

 1
 11

 7
 192

 8
 139

 2
 53

 3
 6

 2
 2

 2
 5

 2
 5

 20
 305

 17
 242

 3
 63

 40
 672

 15
 299

 24
 369

 1
 4

 46
 287

 44
 258

 1
 3

 1
 3

 1
 1

 41
 251

 2
 29

 2
 29

 1
 9

 1
 9

 1
 9

 162
 2986

 1
 23

 1
 23

 161
 2963

 8
 205

 9
 154

 3
 19

 9
 113

 49
 604

 1
 2

 1
 3

 1
 27

 13
 234

 1
 13

 1
 16

 9
 211

 1
 10

 5
 84

 1
 3

 1
 16

 2
 3

 2
 20

 2
 28

 7
 457

 3
 27

 1
 17

 1
 9

 1
 13

 1
 5

 1
 11

 2
 13

 1
 3

 9
 240

 1
 13

 6
 70

 8
 320

 43
 354

 2
 19

 2
 19

 10
 44

 7
 32

 1
 2

 2
 10

 3
 43

 2
 30

 1
 13

 28
 248

 2
 43

 24
 202

 2
 3

 5
 31

 4
 29

 2
 27

 2
 27

 1
 1

 1
 1

 1
 1

 1
 1

 1
 2

 1
 2

 1
 2

 50
 222

 50
 222

 1
 1

 1
 1

 44
 200

 6
 34

 1
 1

 1
 5

 1
 2

 1
 1

 3
 12

 5
 13

 1
 2

 5
 14

 2
 13

 2
 10

 1
 1

 1
 1

 4
 16

 2
 15

 2
 5

 1
 2

 1
 27

 1
 12

 3
 14

 5
 21

 5
 21

 8
 13

 1
 2

 1
 2

 1
 2

 5
 9

 2
 3

 1
 1

 1
 2

 1
 1

 1
 1

 1
 1

 1
 1

 1
 4

 1
 4

 1
 1

 1
 1

 1
 1

 1
 1

 1
 1

 1
 1

 3
 5

 3
 5

 2
 4

 2
 4

 2
 4

 1
 1

 1
 1

 1
 1

 5
 50

 5
 50

 5
 50

 5
 50

 5
 50
